# The Role of Ferroptosis in Acute Respiratory Distress Syndrome

**DOI:** 10.3389/fmed.2021.651552

**Published:** 2021-05-07

**Authors:** Mengdi Qu, Hao Zhang, Zhaoyuan Chen, Xingfeng Sun, Shuainan Zhu, Ke Nan, Wankun Chen, Changhong Miao

**Affiliations:** Department of Anesthesiology, Zhongshan Hospital, Fudan University, Shanghai, China

**Keywords:** ferroptosis, acute respiratory distress syndrome, metabolism, inflammation, oxidative stress

## Abstract

Ferroptosis is a newly discovered type of regulated cell death that is different from apoptosis, necrosis and autophagy. Ferroptosis is characterized by iron-dependent lipid peroxidation, which induces cell death. Iron, lipid and amino acid metabolism is associated with ferroptosis. Ferroptosis is involved in the pathological development of various diseases, such as neurological diseases and cancer. Recent studies have shown that ferroptosis is also closely related to acute lung injury (ALI)/ acute respiratory distress syndrome (ARDS), suggesting that it can be a novel therapeutic target. This article mainly introduces the metabolic mechanism related to ferroptosis and discusses its role in ALI/ARDS to provide new ideas for the treatment of these diseases.

## Introduction

Ferroptosis, a new form of regulated cell death that can be triggered by erastin or RSL3 [(1S,3R)-RSL3], was first reported in 2012 by Dixon (1). Ferroptosis is characterized by the iron-dependent accumulation of lethal levels of lipid peroxides, while the morphology, biology and genetics are obviously different from those of apoptosis, necrosis, autophagy, and other forms of cell death (1). Amino acid, iron and lipid peroxide metabolism and other metabolic processes are closely related to ferroptosis (2). Studies have shown that ferroptosis, as the main cause of organ damage-related cell death, is involved in many pathological processes, such as neurodegenerative diseases, cancer and ischemia-reperfusion injury (2, 3).

Acute lung injury (ALI), resulting from both direct (e.g., pneumonia) and indirect (e.g., sepsis) pulmonary injuries, refers to pulmonary edema and atelectasis caused by diffuse alveolar-capillary injury and is characterized by refractory hypoxemia and pulmonary infiltration (4). Acute respiratory distress syndrome (ARDS) is a serious form of ALI and is described by the 2012 Berlin unified definition (5). The prevalence of ARDS in intensive care units is 10.4%, and there is a high mortality rate (35–46%) (6) but a lack of effective treatments (7).

In recent years, the role of ferroptosis in ARDS has been gradually revealed, and increasing attention has been given to the importance of regulating ferroptosis in the treatment of ARDS.

## Major Metabolic Mechanisms of Ferroptosis

**Figure 1:**
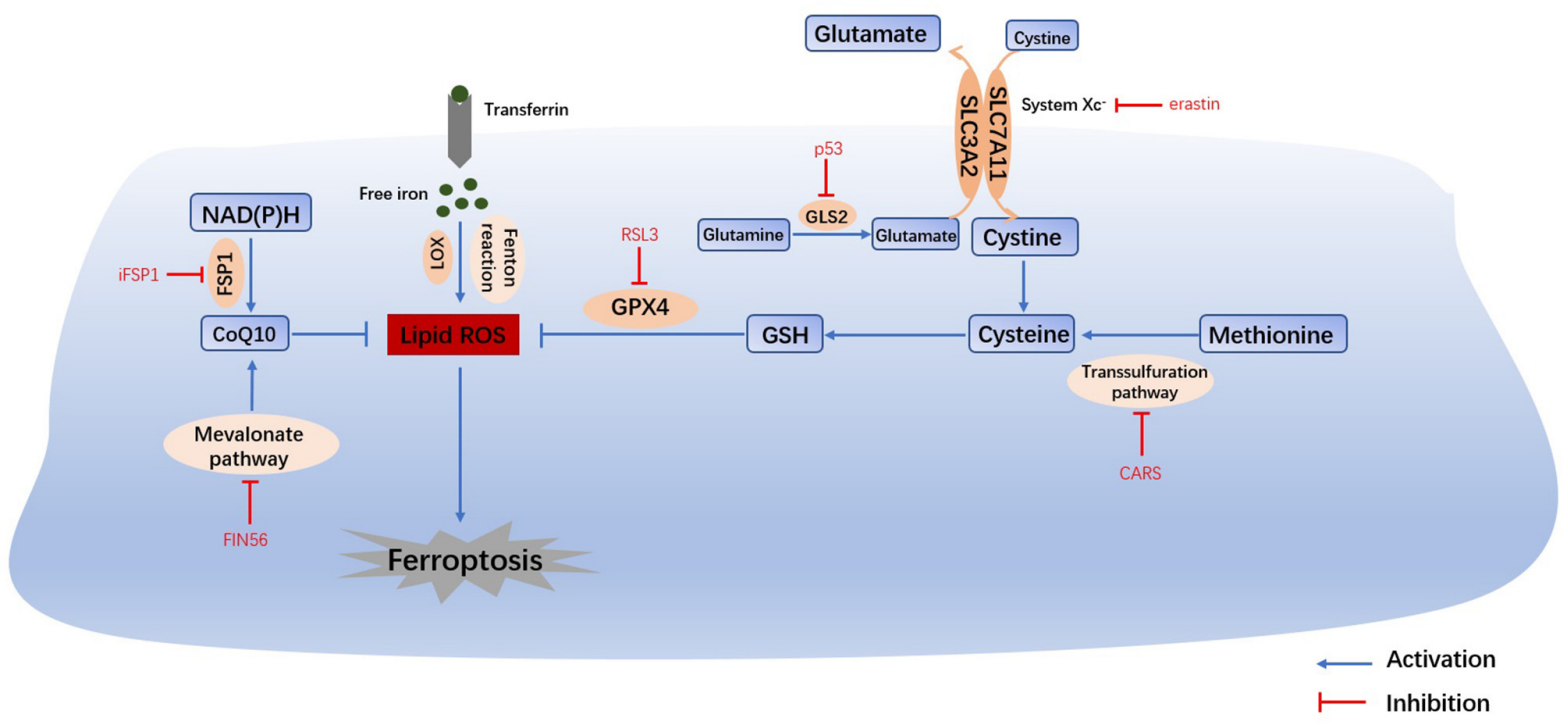
Main mechanisms of ferroptosis. The Fenton reaction, LOX and PUFAs facilitate the generation of lipid ROS. Cysteine can be generated from the uptake of cystine via system Xc- or the transsulfuration pathway. Amino acid metabolism and NAD(P)H, suppresses the synthesis of GSH and CoQ10, thus inhibiting the reduction in lipid ROS. The accumulation of lipid ROS leads to ferroptosis. Therefore, iron homeostasis, lipid peroxidation and amino acid metabolism are the main regulators of ferroptosis. System Xc-, cystine/glutamate transporter; GLS, glutaminase; GSH, glutathione; GPX4, glutathione peroxidase 4; LOX, lipoxygenase; FSP1, ferroptosis suppressor protein 1; CoQ10, coenzyme Q10.

Ferroptosis is a form of cell death that is regulated by multiple genes and involves multiple metabolic processes, such as iron homeostasis, amino acid metabolism and lipid peroxidation. The mechanism is very complex as is shown in Figure 1, and it will be better explained in the following aspects.

## Iron Metabolism

Iron overload is one of the key events in ferroptosis. Iron is necessary for the accumulation of lipid peroxides, and iron ingestion, storage and transport all affect ferroptosis (2). Iron homeostasis is regulated by a series of iron regulatory proteins (IRPs). Extracellular iron enters the cell through transferrin (TF) and its receptors, and then Fe^2+^ can produce lipid peroxides via the Fenton reaction or the iron-containing enzyme lipoxygenase (LOX) (8). Most intracellular Fe^2+^ is stored in ferritin (FT), and so there is very little free Fe^2+^ (9). The degradation of FT increases the level of intracellular Fe^2+^, enhances lipid peroxidation, and induces ferroptosis. This process is related to autophagy and is regulated by nuclear receptor coactivator 4 (NCOA4) (8). Iron response element binding protein 2 (IREB2) (1) and other proteins related to iron metabolism (2) (HSPB1, CISD1, etc.) can also increase the sensitivity of cells to ferroptosis.

## Amino Acid Metabolism

Glutathione (GSH) depletion is another key event in ferroptosis. The cystine/glutamate antiporter System Xc-, which is mainly composed of SLC3A2 (solute carrier family 3 member 2) and SLC7A11 (solute carrier family 7 member 11) (10), is located on the cell membrane and transports extracellular cystine and intracellular glutamate at a ratio of 1:1. Extracellular cystine and intracellular cysteine are essential for the biosynthesis of GSH. Cystine ultimately generates GSH through a series of enzymatic reactions, and GSH is the essential substrate for glutathione peroxide enzyme 4 (GPX4) to degrade phospholipid hydroperoxide (PLOOH) (11). GPX4 is at the intersection of GSH metabolism and lipid peroxidation, both of which are related to ferroptosis. Downregulation of SLC7A11 can also lead to ferroptosis through a decrease in GPX4 activity (12). In addition, methionine can transfer sulfur atoms to serine to generate cysteine through the transsulfuration pathway, which can be upregulated by the knockout of cysteinyl-tRNA synthetase (CARS), thus making cells resistant to ferroptosis (13).

Under physiological conditions, a high level of extracellular glutamate can inhibit the activity of System Xc- and prevent the uptake of cysteine (14). Therefore, glutamate is a natural trigger for ferroptosis and has the same effect as erastin and other System Xc- inhibitors (8). In addition, glutamine is abundant in tissue and plasma and can be converted into glutamate by glutaminase (GLS1 and GLS2) catalysis. Glutaminolysis is necessary for the tricarboxylic acid cycle and lipid biosynthesis, and α-ketoglutarate, as the product of decomposition, is involved in ferroptosis (15). Therefore, when glutamine is deficient or its decomposition is inhibited, reactive oxygen species (ROS) accumulation, lipid peroxidation and ferroptosis are also inhibited. Furthermore, GLS2, as a target gene of the tumor suppressor p53, is closely related to ferroptosis (16). In summary, the metabolism of amino acids (especially glutamate and cystine) plays an important role in the pathological process of ferroptosis.

## Lipid Metabolism

The most prominent feature of ferroptosis is plasma membrane damage caused by the production of iron-dependent lipid peroxides (lipid ROS) (8). ROS include products of oxygen reduction, such as O^2−^, H_2_O_2_ and ·OH. Oxygen homeostasis is crucial to normal cellular functions, and the abnormal accumulation of ROS is harmful to the body (3). During ferroptosis, the reduction reaction mediated by GPX4 and ferroptosis suppressor protein 1 (FSP1, formerly known as mitochondrial apoptosis inducing factor 2, AIFM2) is inhibited, and the oxidation reaction catalyzed by Fe^2+^ and a series of iron-dependent enzymes (mainly LOX) is enhanced, inducing the accumulation of polyunsaturated fatty acids (PUFAs) (8). Then, lipid peroxidation drove by PUFAs increases the permeability of the cell membrane and makes the cell more sensitive to oxidation, which eventually leads to ferroptosis (17, 18). The inhibition of lipid peroxidation and the consumption of PUFAs can inhibit ferroptosis (2).

## The FSP1-NAD(P)H Pathway

Bersuker and Doll found that FSP1 and GPX4 had a strong synergistic effect (19, 20). In the FSP1-NAD(P)H pathway, coenzyme Q10 (CoQ10) can reduce lipid peroxidation by inhibiting the accumulation of free radicals, and FSP1 catalyzes the production of CoQ10 through NAD(P)H. iFSP1, an inhibitor of FSP1, can induce ferroptosis in cells that overexpress FSP1 (20). In conclusion, the FSP1-CoQ10-NAD(P)H pathway cooperates with GPX4 and GSH to inhibit lipid peroxidation and ferroptosis. Moreover, CoQ10 can also be generated by the mevalonate (MVA) pathway. FIN56 can not only accelerate the degradation of GPX4 but also consume CoQ10 by affecting the MVA pathway, ultimately leading to excessive lipid peroxide accumulation and ferroptosis (21).

## Pathogenesis of ARDS

### Pathological Mechanism of ARDS

The most common cause of ARDS is bacterial or viral pneumonia, while sepsis, severe trauma and gastric reflux and aspiration are also common factors (22). The inflammatory response is activated by infection, trauma, or damage to the lung. Moderate inflammation is conducive to the clearance of pathogens, but excessive inflammation may lead to alveolar damage and increased permeability of the pulmonary capillary endothelium and alveolar epithelium, after which protein-rich fluid exudes from the alveolar cavity, leading to pulmonary edema (22). Therefore, ARDS is the pulmonary manifestation of systemic inflammatory response syndrome (SIRS) (23), which involves various inflammatory cells (macrophages, neutrophils, vascular endothelial cells, and platelets), and the inflammatory mediators and cytokines released by these cells indirectly mediate inflammation in the lung.

The levels of proinflammatory cytokines (IL-1β, IL-8, TNFα, TGFβ1, etc.) are very high in the pulmonary edema fluid in ARDS patients, and cytokines can activate the innate immune system. Activated neutrophils can produce toxic substances such as ROS and proteases, leading to pulmonary endothelial and alveolar epithelial damage and even necrosiss (24). Necrosis and the accumulation of edema fluid, in turn, trigger more severe inflammation and immune responses. Many clinical trials have evaluated the potential effect of anti-inflammatory therapy to treat ARDS (25–27). In summary, excessive inflammation and increased permeability of the pulmonary capillary endothelium and alveolar epithelium lead to alveolar damage, which is the main pathological mechanism of ARDS.

### Iron Overload

Various cell types in the lung, including epithelial cells and macrophages, can produce iron metabolism-related proteins to regulate iron homeostasis and protect lung tissue from oxidative stress (28). Iron metabolism disorders are closely related to lung tissue damage in ARDS patients (29, 30); that is, too much iron can generate ROS and cytotoxicity through the Fenton reaction. Many clinical studies have shown that the severity of ARDS is associated with the levels of iron and iron-related proteins (31). One study indicated that iron in blood products leads to an increase in iron in blood recipients, which promotes the occurrence of blood transfusion-related ALI (32). Elevated levels of Fe^2+^ and iron regulators, such as TF and FT, can be detected in the bronchoalveolar lavage fluid (BALF) of ARDS patients (28, 29, 33–35). In an oleic acid-induced ALI model in mice, iron overload was detected in the lung tissue (36). Moreover, supplementing mice with iron in advance exacerbates damage to the lung (12). A recent study also showed that increased apoptosis in mice with iron overload exacerbated ALI. However, this effect was quite transient and did not affect the degree of inflammation or speed of recovery in ALI (37). Ferroptosis is an iron-dependent process, and iron overload is the driving factor of it. In ARDS, iron overload leads to ferroptosis, which aggravates lung injury. In other words, the cells appear to be overloaded with iron due to ferroptosis and the disease becomes increasingly worse. Therefore, we have enough reason to believe that ferroptosis plays a crucial role in ARDS. And whether iron overload actually causes lung injury or is just a byproduct of ferroptosis remains to be confirmed.

### Oxidative Stress

Exhaled breath analysis is expected to be clinically used for the early diagnosis and prediction of ARDS, and most candidate markers are related to oxidative stress (38). Oxidative stress causes damage to the barriers of the pulmonary epithelium and endothelium, and neutrophils accumulate in large quantities in the alveolar fluid, producing proinflammatory cytokines and ROS. Moreover, ROS can further increase the level of cytokines, exacerbating tissue damage and edema. Therefore, oxidative stress plays an important role in the pathogenesis of ARDS (39, 40). ROS are known as important mediators of ARDS (41–44), and enzymes related to the production of ROS (xanthine oxidase (XOR) (45), endothelial nitric oxide synthase (eNOS) (46), cytochrome P450 (CYP) (7), and NADPH oxidase (NOX) (47)) have been reported to be involved in ARDS. The level of malondialdehyde (MDA), a product of lipid peroxidation, is increased in the ALI mouse model (36, 48). In fact, MDA is commonly regarded as a marker of ferroptosis. Increases in both neutrophils and ROS can be detected in ARDS patients (49).

GSH is the most important antioxidant in the airway epithelium and exerts antioxidant effects through the removal of ROS (50) and the repair of cellular damage (51), thus helping to alleviate inflammation (52). Decreased GSH and increased oxidized GSH (GSSG) were observed in both ALI patients and animal models (12, 36, 42). A lack of GSH in alveolar fluid made ARDS patients more susceptible to lung injury (49). Moreover, ROS and GSH metabolism is the main feature of ferroptosis. Whether these metabolic processes are also involved in the pathogenesis of ARDS by regulating ferroptosis needs to be investigated. Inhibition of ferroptosis can inhibit the production of these peroxides, thus reducing the severity of ARDS, which is a potential therapeutic strategy.

### Role of Ferroptosis in ARDS

Indicators related to ferroptosis were detected in ALI animal models, including increased Fe^2+^, ROS, MDA and decreased GSH. And inhibitors of ferroptosis have the potential to alleviate lung damage. These results show that ferroptosis is indeed associated with ARDS. However, the specific mechanism by which ferroptosis affects the onset of ARDS is still unclear.

As a clinically common respiratory disease, the main pathogenic mechanism of ARDS is the apoptosis of alveolar epithelial cells and pulmonary microvascular endothelial cells and the polarization of alveolar macrophages. Then, a large amount of ROS and inflammatory factors trigger an imbalance between the oxidation and antioxidant systems, and the “cytokine storm” leads to the disturbance in the local microenvironment of the lungs, resulting in a series of inflammatory reactions (53). Unlike apoptosis, ferroptosis is associated with a consistent release of damage-associated molecular patterns (DAMPs) and inflammatory cytokines, which promote a series of inflammatory responses. Therefore, ferroptosis is considered an immunogenic form of cell death (54). Inflammatory cytokines further promote ferroptosis and other forms of cell death, thus forming a self-amplifying loop that mutually promotes organ damage (55). Ferroptosis plays a key role in ALI in mice, and ferroptosis inducers can exacerbate pulmonary edema and alveolar inflammation, accompanied by high levels of cytokines (IL-1β, IL-6, and TNF-α), while these effects can be reversed by ferroptosis inhibitors (12, 41, 48). In the latest study (36), ARDS animal model was prepared by injecting oleic acid into the tail vein of mice. The results showed that the pulmonary cells of ARDS group showed mitochondrial shrinkage and rupture of the mitochondrial membrane. In addition, iron overload, GSH depletion and down-regulated expression of ferritin appeared in lung tissues. Similar results were observed in the model of lung ischemia-reperfusion injury (56). The above results suggest that ferroptosis is involved in the pathogenesis of lung injury, which will provide a new theoretical basis for the clinical treatment of ARDS. However, no clinical studies have examined the association of these ferroptosis indicators with severity and prognosis of ARDS.

Ferroptosis is one of the critical mechanisms contributing to sepsis-induced injuries in mice models, including heart, liver, intestine, and the inhibition of ferroptosis via enhancing GPX4 or nuclear factor erythroid 2-related factor 2 (Nrf2) alleviates these injuries (57–60). It contradicts that erastin attenuates the inflammatory response, resulting in inhibition of sepsis development (61). So what is the real role of ferroptosis? We know that sepsis is also an important inducer of ARDS, so it is natural to consider the role of ferroptosis in sepsis-induced lung injury. Furthermore, the lipid peroxidation in ferroptosis drives pyroptosis, indicating a crosstalk between ferroptosis and other forms of cell death in sepsis, and such interactions may also exist in ARDS (62).

**Figure 2:**
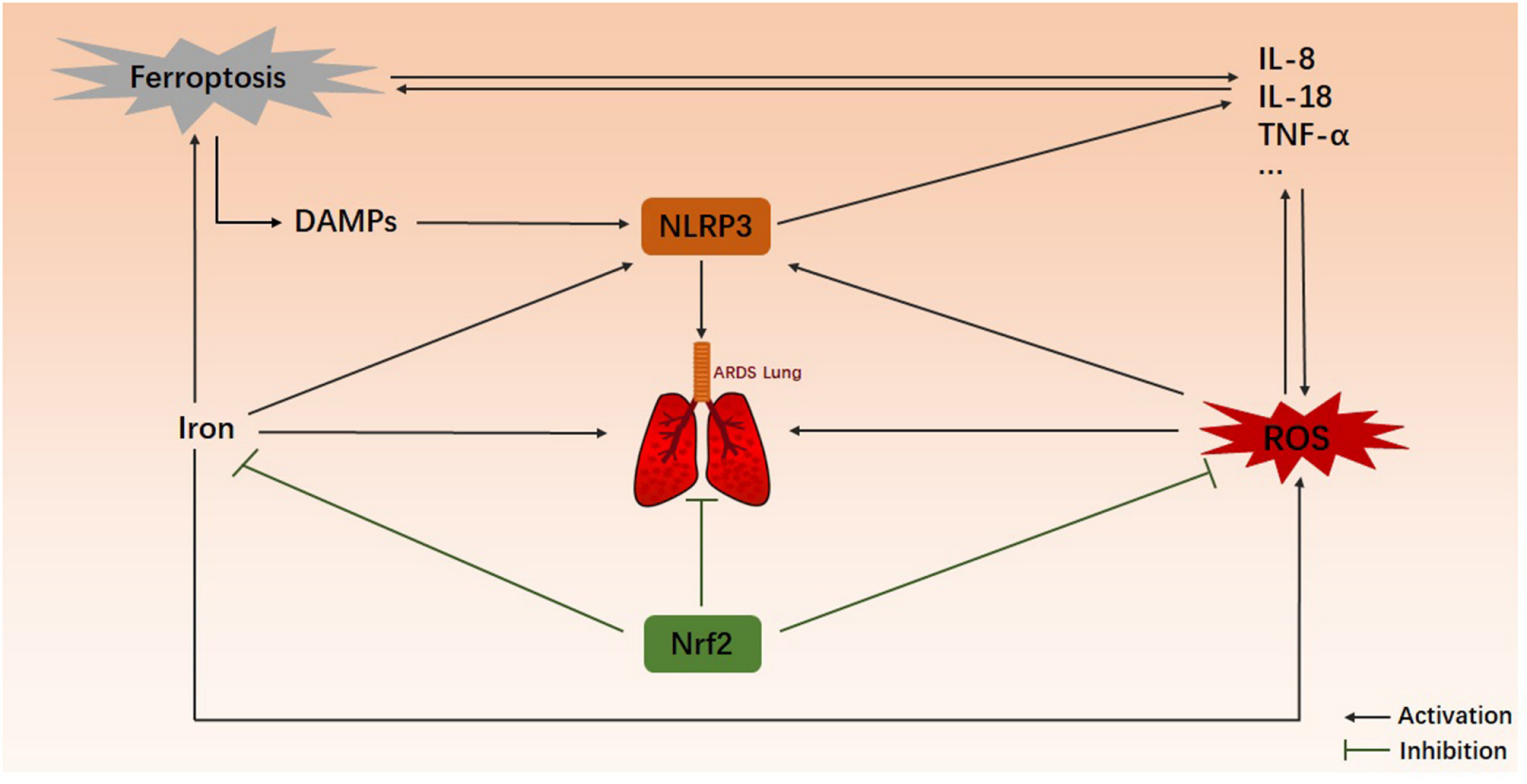
Possible relationship between ferroptosis and ARDS. Here is an ARDS lung. DAMPs, ROS, and iron overload contribute to ARDS, and they can activate the NLRP3 inflammasome, which promotes the maturation and secretion of inflammatory factors, forming a self-amplifying loop with ferroptosis. Nrf2 inhibits the generation of ROS and iron to negatively regulate ferroptosis. However, the direct link between ferroptosis and ARDS is unclear. DAMPs, damage-associated molecular patterns; ROS, reactive oxygen species; NLRP3, NOD-like receptor protein 3; Nrf2, nuclear factor erythroid 2-related factor 2.

Ultimately, ferroptosis causes cellular injury primarily through inflammation and oxidative stress, and the NOD-like receptor protein 3 (NLRP3) inflammasome and Nrf2 are key molecules in these processes (12, 41, 42, 63, 64). Both of them are important regulatory molecule in ARDS and can be used as targets for the treatment of ARDS (65–71). Of course, they could be recognized as mediators of ferroptosis in ARDS, as shown in Figure 2. Ferroptosis may promote inflammation and swelling of alveolar epithelial cells via the NLRP3 inflammasome, bringing about ARDS. NLRP3 is a key mediator in the process of pyroptosis, so crosstalk between ferroptosis and pyroptosis may occur in the pathogenesis of ARDS, aggravating lung injury. Further animal experiments and clinical studies are needed to verify these points. The regulation of the NLRP3 inflammasome by inhibiting ferroptosis to thereby alleviate ARDS may also be a new therapeutic strategy.

At the same time, recent studies have shown that Nrf2 inhibits ferroptosis by regulating the expression of SLC7A11 and heme oxygenase-1 (HO-1), thus alleviating lung injury (12, 64). Nrf2 activators can cause a reduction in ROS and prevent GSH depletion and lipid peroxide accumulation. As a result, ferroptosis is inhibited, thereby alleviating ALI and producing the same effect as that of Fer-1 (42). In addition, inhibitor of apoptosis-stimulating protein of p53 (iASPP) could inhibit ferroptosis and ALI by upregulating Nrf2. Furthermore, the levels of a variety of proinflammatory cytokines (TNF-α, IL-1β, and IL-6) were also decreased (41).

In balance, most studies have not directly focused on the relationship between ferroptosis and ARDS. Therefore, the direct relationship between them needs to be explored, together with more treatments targeting ferroptosis.

### Ferroptosis Applications in ARDS Therapy

**Figure 3:**
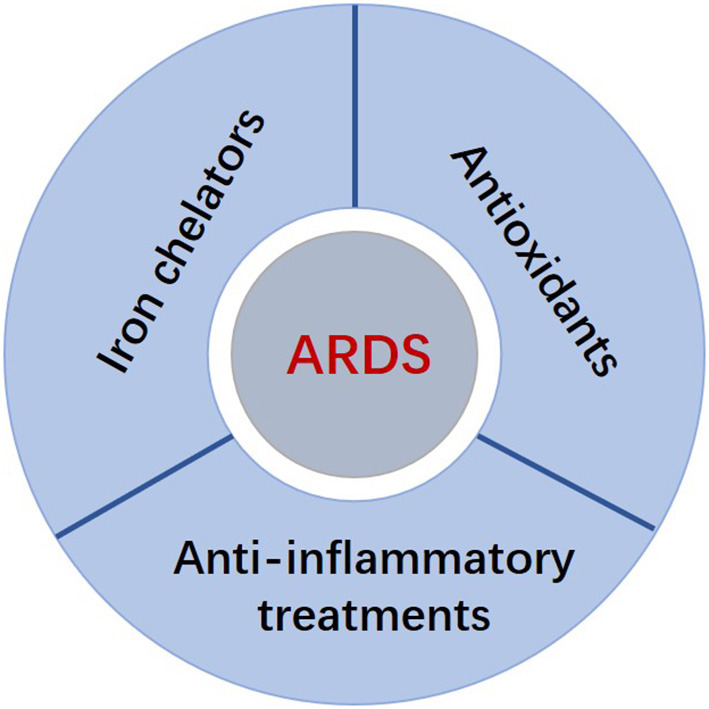
Treatments for ARDS. Therapeutic targets for ARDS associated with ferroptosis, such as iron chelators, antioxidants and anti-inflammatory treatments.

Disorders of iron homeostasis, the depletion of GSH, and oxidative stress are the key points leading to ferroptosis and could be used as targets for the treatment of ARDS (Figure 3).

### Iron Chelators

Iron chelators (deferoxamine, deferiprone, and deferasirox), especially deferoxamine (DFO), have been approved by the FDA for the treatment of iron overload (72). In various animal models of infection, DFO has immunomodulatory effects to resist pathogens such as bacteria, viruses, and fungi, in addition to chelating iron (73). DFO can reduce the levels of inflammatory cytokines and ROS *in vitro*, exerting anti-inflammatory effects (74). DFO inhalation was shown to improve pulmonary fibrosis and prevent a decline in pulmonary function in mice (75). Simultaneous perfusion of DFO and FT could attenuate leakage syndrome in isolated mouse lungs (76). In summary, iron chelators may also be effective in treating ARDS, and the mechanism may be related to the suppression of ferroptosis.

### Antioxidants

Antioxidants can reduce the severity of ARDS (7). Several kinds of drugs decrease the levels of lipid peroxidation and ROS, attenuate inflammation and oxidative stress, and ultimately alleviate ARDS in mice and improve gas exchange (77–80). GSH supplementation could significantly alleviate mitochondrial dysfunction and oxidative damage in the LPS-induced ALI model (81). Animal experiments and clinical studies have shown that regulating the level of GSH (82) via N-acetylcysteine (NAC) could promote the production of GSH and alleviate ALI (7). NAC treatment resulted in increased pulmonary compliance and reduced pulmonary edema (83). In New York, two patients with ARDS caused by COVID-19 showed significant relief of dyspnea after oral and intravenous GSH supplementation, demonstrating that this is indeed a new treatment strategy for ARDS (52). Given the importance of GSH in ferroptosis, it is also worth investigating whether GSH plays a role in the treatment of ARDS by inhibiting ferroptosis.

### Anti-inflammatory Treatments

Inhibiting inflammation is an important treatment strategy for ARDS. Combined inhibition of ferroptosis and inflammation has been reported to treat a variety of diseases, such as stroke, myocardial infarction, and pancreatitis (54, 55). In ALI mouse models, ferroptosis inhibitors reduced inflammatory cytokines and pulmonary edema to treat ALI (41, 48). The exact mechanism of ferroptosis and inflammation needs to be confirmed by additional experiments, and ways to modulate inflammation by controlling ferroptosis also need to be further explored. These studies will provide new strategies for the clinical treatment of ARDS.

### Perspective

Ferroptosis is a newly discovered form of cell death, and ferroptosis regulators provide new therapeutic directions for many refractory diseases (84). Ferroptosis is an abnormal metabolic process involving iron, lipids and amino acids, and the metabolism of these substances plays a key role in cell proliferation and differentiation. Ferroptosis is characterized by metabolic imbalances and disturbances in redox homeostasis, in which the metabolic process is not independent, but a part of a complex metabolic network. The results of animal experiments and clinical trials preliminarily show that a variety of diseases and pathological processes are closely related to ferroptosis, and intervention in ferroptosis can effectively delay the progression of the disease and improve clinical symptoms to a certain extent. However, research on ferroptosis is still in its infancy. Studies on ferroptosis and lung cancer have made some progress, and ferroptosis inducers as new adjuvants based on traditional treatments have shown their effectiveness. The development of new ferroptosis inducers and the application of multiple forms of combined treatment strategies may be expected to provide new ideas for the treatment of lung cancer.

Recent studies have shown that ferroptosis is closely related to ALI/ARDS, making it a potential target for the treatment of ALI/ARDS. The current studies are based on animal models, while there is a lack of clinical studies. In this context, it is worth noting that the precise role of ferroptosis in the development of ALI/ARDS, and the pharmacological inhibition of ferroptosis, but not necroptosis or apoptosis, protects lung tissues from injury, which remains to be fully elucidated. Considering that ferroptosis was proposed as a brand new concept, there are still large gaps that need to be filled. Clinically, whether the key molecules of ferroptosis can be used as biomarkers to predict the severity of ARDS are needed to investigate. Also, it is necessary to prove whether ferroptosis is the core mode of cell death in ARDS and their crosstalk mechanism.

## Author Contributions

MQ and HZ: conception and design. All authors: manuscript writing and Final approval of manuscript.

## Conflict of Interest

The authors declare that the research was conducted in the absence of any commercial or financial relationships that could be construed as a potential conflict of interest.
